# Effect of Multi-Pass Power Spinning on Microstructure Homogenization and Mechanical-Property Strengthening of Ti_2_AlNb-Based Alloy Using Welded Tube Blank

**DOI:** 10.3390/ma15031013

**Published:** 2022-01-28

**Authors:** Sibing Wang, Wenchen Xu, Bo Wang, Guoping Yang, Debin Shan

**Affiliations:** 1 School of Materials Science and Engineering & National Key Laboratory for Precision Hot Processing of Metals, Harbin Institute of Technology, Harbin 150001, China; wangsibing@hit.edu.cn; 2 Department of Technology Development, Beijing Spacecrafts, Beijing 100094, China; wangb529@126.com; 3 Capital Aerospace Machinery Co., Ltd., Beijing 100076, China; pacoyang_2005@163.com

**Keywords:** Ti_2_AlNb-based alloy, power spinning, microstructure, texture, mechanical property

## Abstract

Long seamless tubes of Ti_2_AlNb-based alloys are difficult to manufacture through conventional forming methods. In this study, a multi-pass power spinning process was first utilized to fabricate thin-walled tube of Ti-22Al-24Nb-0.5Mo alloy using welded thick tube blank, assisted by on-line electro-magnetic induction heating to maintain high spinning temperature during the whole spinning process. After six-pass hot power spinning at 950 ± 30 °C, the microhardness difference of BM (base metal), HAZ (heat affect zone) and FZ (fusion zone) became much smaller, and the microhardness fluctuation ΔHV dropped to 32 from 122 of the as-welded joint due to the phase composition and microstructure homogenization. The grain size of B2 phase was refined to 0.4/0.6 μm from 2.7/10.8 μm of the as-received BM/FZ, respectively. Meanwhile, the B2 phase <111>_B2_//ND texture of the as-received rolled sheet weakened during multi-pass spinning due to recrystallization, which co-existed with <001>_B2_//ND texture in final pass. The ultimate tensile strength in axial/tangential direction was increased to 1245/1299 MPa from 1206/1010 MPa of the as-received rolled sheet, respectively, mainly due to the effect of fine grain strengthening. This study provides an effective way to manufacture high-performance tubular workpieces with low cost and high efficiency.

## 1. Introduction

Based on the superior properties, such as high specific strength, outstanding oxidation and splendid creep resistance [1,2,3,4], the Ti_2_AlNb-based alloys have presented amounts of brilliant performances in aviation and aerospace field, and the corresponding thin-walled tubular workpieces exhibit increasing application potential in aviation and automotive industries [5,6]. Especially, during the long-term service, the temperature of the aviation components can reach 650 °C or even 750 °C [5,6], which make the Ti_2_AlNb-based alloys good candidates for high-temperature load-bearing structural components. As typical multiple phase alloys, the Ti_2_AlNb alloys have quite a few microstructural morphologies [7,8,9,10]. Usually, the Ti_2_AlNb alloys consist of the O phase (Cmcm symmetrical structure, Ti_2_AlNb), the bcc B2 phase (ordered Pm3m symmetric structure)/β phase (disordered structure), and the hcp α_2_ phase (DO19 P63/mmc symmetrical structure, Ti_3_Al) [1]. Based on the Boehlert research [11], the tensile elongation of Ti-22Al-25Nb fluctuated between 0 and 16% at room-temperature, and the yield strength could be manipulated between 650 and 1600 MPa [12,13]. Du et al. [14] reported that the tensile strength of the multilayer structure of Ti_2_AlNb-based alloy decreases from 981 MPa to 684 MPa at 650 °C, because of the precipitation of coarsened O laths during furnace cooling.

As considering the mechanical property, i.e., high strength accompanies low ductility, the as-rolled Ti_2_AlNb-based alloy sheets are usually formed into complex-shaped workpieces by hot forming at high temperature over 900 °C, and even higher than 950 °C or 970 °C [14]. Besides, superplastic forming and hot gas forming were used to produce the complex-shaped components of Ti_2_AlNb-based alloys, such as Ti-22Al-24Nb box-shaped component [15] and cup-shaped Ti-22Al-24.5Nb-0.5Mo component [16]. However, no study on hot forming of long thin-walled tubes of Ti_2_AlNb-based alloys has been reported until now. It is well known that power spinning is an outstanding process to manufacture thin-walled tubular components with high performance [17,18]. However, the large-diameter tube billet of Ti_2_AlNb-based alloys for power spinning is quite difficult to prepare through conventional extrusion and ring forging because of their inferior hot workability. The combination of hot pressing and welding from sheet plate to form Ti_2_AlNb-based alloy tubes shows high production efficiency and low cost, while the poor weldability deteriorates the strength and ductility of welded tubes of Ti_2_AlNb alloys. As Chen et al. reported, the Ti-22Al-25Nb EBW (electronic beam welding) joints exhibited no ductility at 650 °C and failed at 333–387 MPa in fusion zone (FZ) as the inter granular failure, indicating especially infirm B2 phase grain boundaries in the FZ [12]. Li et al. reported that the full-penetrated EBW weld joint of thick plate suffered about 1000 MPa tensile stress in the center of the FZ, resulting in the risk for cracking [19]. Compared with welding sheets, welding tubes need more complex clamping fixture and welding process, while the strength and ductility reduced more significantly [20]. In addition, there is no research concerning power spinning of thin-walled tubes of Ti_2_AlNb-based alloys available to date. Therefore, it is difficult to produce thin-walled Ti_2_AlNb-based tubes with satisfied shape and admirable mechanical property by way of tube welding and power spinning process.

In this study, a multi-pass hot power spinning process using welding thick tube blanks of Ti-22Al-24Nb-0.5Mo alloy was first designed and performed to form thin-walled tubular parts without cracks successfully. The microstructure evolution and mechanical properties of base metal and EBW joint during hot spinning were investigated, which exhibited a homogenized strengthening effect with the increase in thickness reduction. This work is expected to provide a feasible way to fabricate high performance thin tubular workpiece of Ti_2_AlNb alloys with low cost and high efficiency.

## 2. Experimental Material and Method

### 2.1. Material Selection and Experimental Procedure

**Figure 1:**
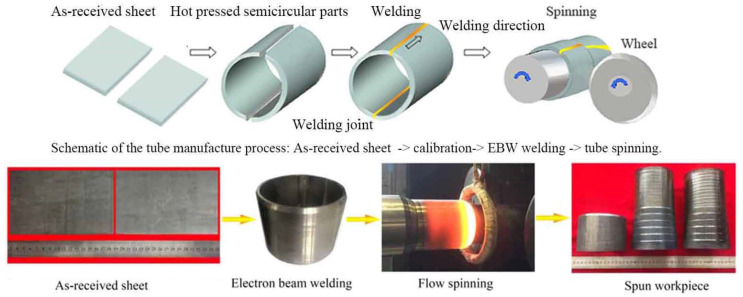
Schematic of hot power spinning assisted by on-line electro-magnetic induction heating.

The hot rolled Ti-22Al-24Nb-0.5Mo (at%) sheets with 6 mm in thickness were provided by IMR (Institute of metal research, Chinese Academy of Sciences). The dimension of the pre-spun welded tube was 100 mm (diameter) × 6 mm (thickness) × 80 mm (length) in this study. To make welded tubes, two pieces of as-received Ti_2_AlNb sheets were hot pressed to semicircular parts at 920 ± 30 °C, and then connected by electronic beam welding (EBW). The spun tube workpiece was formed by 6-pass hot flow spinning under the temperature 950 ± 30 °C. Subsequently, the spun workpiece was cooled in the air, followed by annealing treatment at the temperature 800 °C for 3 h, to relieve the residual stress. Figure 1 shows the whole manufacture process.

### 2.2. Temperature Control System

**Figure 2:**
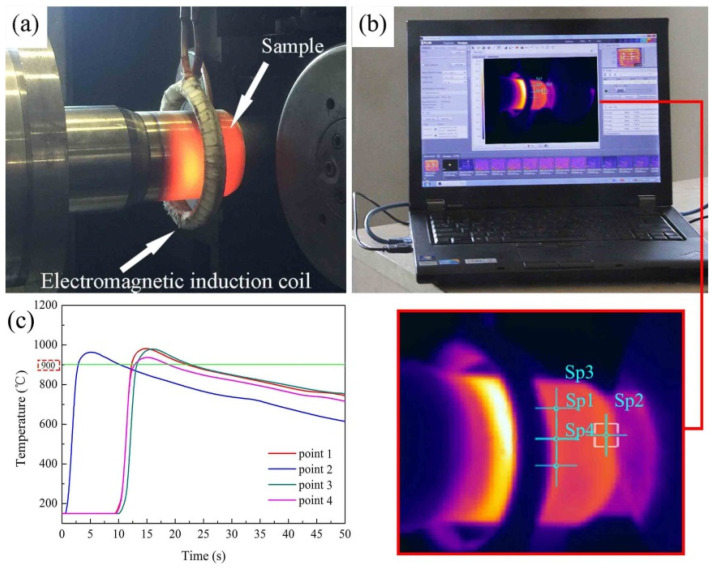
The apparatus of electromagnetic induction heating system for hot power spinning: (**a**) electromagnetic induction heating, (**b**) infrared temperature monitoring system, (**c**) and time-temperature curve during hot power spinning.

The ductility exhibited high correlation with the temperature as well as strain rate closely of the Ti_2_AlNb-based alloy, as reported by our previous research [21]. In hot spinning of Ti_2_AlNb alloy, the spinning temperature should be kept relatively stable in 900–1000 °C. In this study, the temperature control system was designed to heat tube billet and control the temperature change during hot spinning, as shown in Figure 2, which consisted of an electromagnetic induction heating device and an infrared temperature monitoring system, (FLIR Systems, Inc., Arlington, WA, USA). During hot spinning, the electromagnetic induction coil moved synchronously with the roller along axial direction to heat tube billets, 950 ± 30 °C. Before the onset of the hot spinning process, the preheating temperature of the mandrel and tube billets should be higher than 650 °C and 750 °C, respectively.

### 2.3. Spinning Process Scheme

The spinning experiments were performed on a CNC spinning machine with two rollers symmetrically distributed around the mandrel. Backward flow spinning method was adopted in this study. During tube spinning, the thinning ratio per pass *ψ*_i_ was chose as 20%. The total thickness reduction (*ψ*_f_ = (*t*_0_ − *t*_f_)/*t*_0_, where *t*_0_ was the initial wall thickness of the tubular blank and *t*_f_ was the final wall thickness of the spun parts), was chosen as 74% after 6 passes. Table 1 listed the main process parameters of flow spinning used in the spinning experiment. The strain rate in the present hot power spinning experiment was ~0.2 s^−1^ according to the following equation [22].

(1) $${\dot{\varepsilon }}_{e}=\frac{2}{\sqrt{3}}\frac{v_{0}\sin\alpha_{\rho }}{t_{0}{\left(1-\psi_{t}\right)}^{2}}$$

*v*_0_ = *nf* (2)

where *v*_0_ was feed speed, α_ρ_ was front angle of roller, *ψ*_t_ was reduction, *t*_0_ was initial tube blank thickness, *n* was rotation speed of mandrel, *f* was roller feed rate.

### 2.4. Mechanical-Property Evaluation and Microstructures Characterization

**Figure 3:**
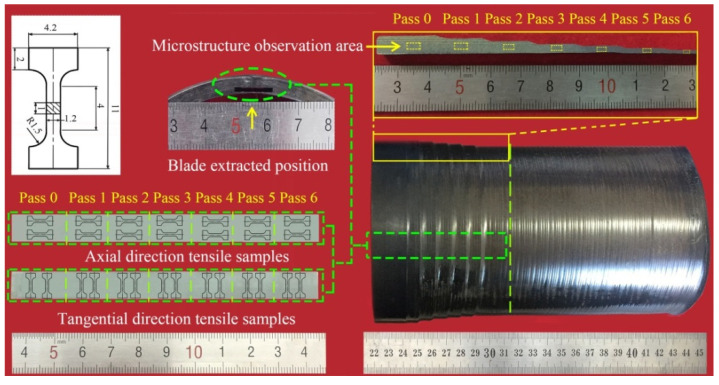
Preparation of microstructure and tensile specimen of axial and tangential direction.

To analyze the mechanical properties of spun tube, tensile specimens with gauge length of 4 mm, thickness of 1.0 mm and width of 1.2 mm were cut along axial and tangential direction from the spun tubes, respectively, see Figure 3. The room temperature tensile test with a strain rate of 1 × 10^−3^ s^−1^ was carried out on an Instron 5569 electronic testing machine, which was repeated five times. Both the axial and tangential tensile tests were carried out on base metal of the multi-pass spun workpiece, see Figure 3. The EBSD analysis was performed on a scanning electron microscope (SEM, Quanta 200FEG) at a step size of 0.1 μm with voltage 30 kV, and post processed with the TSL OIM Analysis 6.1.3 software. The electropolished solution of EBSD samples was perchloric acid, methyl alcohol and butanol (ratio, 6:60:34). The polishing temperature is −20 °C, and the polishing time is 120 s. The microhardness test was carried out on a HVS-1000A microhardness tester (Laizhou Huayin Testing Instrument Co., Ltd., Laizhou, China). A Loading force 1000 g with a dwell time of 15 s was chosen based on ASTM standard E384-99. The TEM observation was carried out on a Talos F200x transmission electron microscope (FEI Company, Hillsborough, OR, USA). The TEM samples were mechanically thinned to 70 μm, and then perforated by ion milling.

Based on SEM, TEM results, the width and length as well as various phase volume fractions were determined (repeated 5 times) by means of the software of Image-Pro Plus, which has often been used to calculate mean values of complicated microstructure [23,24,25].

## 3. Results and Discussion

### 3.1. Initial Microstructures

**Figure 4:**
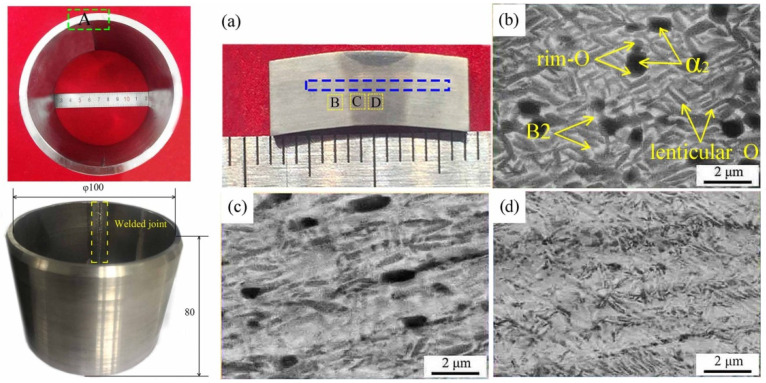
Microstructure of the as-welded joint (SEM micrograph): (**a**) Cross section of the as-welded joint, (**b**) SEM image of zone B (BM) in (**a**), (**c**) SEM image of zone C (HAZ) in (**a**), (**d**) SEM image of zone D (FZ) in (**a**).

The cross-sectional macroscopic morphology of the as-weld joint was presented in Figure 4a. The microstructure of the base metal (BM), B2 + O + α_2_ phases, was depicted in Figure 4b. The area fraction of dark α_2_, grey lenticular O + rim-O, and the light B2 phase was estimated to be 13.2%, 31.4%, 55.4%, respectively, as shown in Table 2.

From BM towards fusion zone (FZ), the area fraction of prior equiaxed α_2_ phase decreased, accompanying with the transformation O → B2. Boehlert et al. [26] systematically analyzed the phase diagram of the Ti-22Al-xNb alloy, indicating that α_2_-Ti_3_Al phase preferred to nucleate earlier than the fine plate-like O phase as the temperature decreased during furnace cooling. Hence, the heat affected zone (HAZ) and BM mainly consisted of B2 + O + α_2_ phases [27].

About 95.5% B2 phase and 4.5% α_2_ phase could be found in FZ due to the huge heat input and the material remelting [28]. As previous researches reported, the phase composition of Ti-22Al-25Nb (at%) alloy was closely related with the cooling rate. If the cooling rate increased higher than 120 K/s, the single B2 phase microstructure tended to occur [29,30]. Boehlert et al. reported that, for Ti-23Al-27Nb (at%) alloy, single B2 phase could be obtained by the way of 1090 °C solution heat-treated and subsequent quench treatment [26]. In addition, due to higher content of Nb element, the transformation of β → a_2_ was impeded effectively since the β phase stabilization were enhanced and higher cooling rate was needed. Thus, the FZ microstructure of Ti-24Al-17Nb (at%) alloy laser-welded-joint was primarily composed of B2 phase [31].

### 3.2. Microstructure Evolution during Multi-Pass Power Spinning

**Figure 5:**
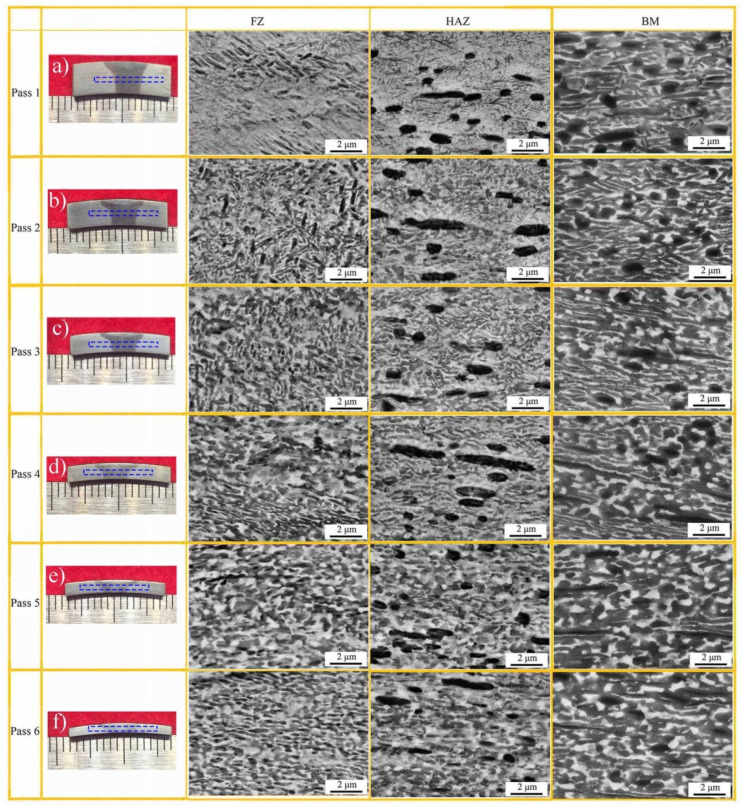
Microstructure evolution of EBW welded joint during 6-pass power spinning: (**a**–**f**) post-welded spun joints with different thinning ratios: 20%, 36%, 49%, 60%, 68% and 74%, respectively.

Figure 5 presents the microstructure evolution of spun tube. The phase fractions of the post-welded spun joints were summarized in Table 2. The lenticular O phase in BM was elongated gradually during 1st to 3rd pass spinning and then dynamically recrystallized during 4th to 6th pass spinning. After 6th pass spinning, the lenticular O phase transformed into global morphology with average diameter ~0.8 μm. The content of O phase fluctuated between 31.4% and 43.8%, and α_2_ phase varied between 13.2% and 12.1% in BM during the whole spinning process.

The microstructure of FZ changed obviously during multi-pass hot spinning, see Figure 5. After 1st and 2nd pass spinning, the content of O phase in FZ reached 47.1% and 47.6%, respectively, and that of α_2_ phase reached 10.3% and 10.7%, respectively, attributed to B2 → B2 + O + α_2_. After 3rd pass spinning, the equiaxed O and α_2_ phases occurred, while the content of O and α_2_ phases only changed little during 3rd to 6th pass spinning.

Different from FZ, the abnormal coarsened a_2_ phase could be seen in HAZ, see Figure 5a–f. Besides, the α_2_ phase content in HAZ changed slightly from 12.8% to 12.1% during the whole process of 6-pass spinning. As Boehlert et al. [26] mentioned, the α_2_ phase occurred in α_2_ + B2 or α_2_ + B2 + O phase region was difficult to be absorbed or transformed completely during the subsequent aging treatment at lower temperature. The rim-O phase was partly accountable over this phenomenon, which impeded the Nb element diffusion between α_2_ and bcc phases [26]. Besides, after 6th pass spinning, the O phase content in HAZ reached 45.7%, which was much greater than that of the as-welded HAZ (5.7%) due to the transformation of B2 → B2 + O + α_2_ at 950 ± 30 °C. Similar to FZ, when the spinning process increased over 3 passes (*ψ*_t_ = 49%), the globalization of O phase in HAZ became quite evident with mean diameter ~0.6 μm after 6th pass spinning. The phase composition and phase content among FZ, HAZ and BM converged gradually with the increase in thinning ratio, as listed in Table 2, due to microstructure homogenization of the post-welded spun workpiece.

#### 3.2.1. Microstructure Evolution of BM

**Figure 6:**
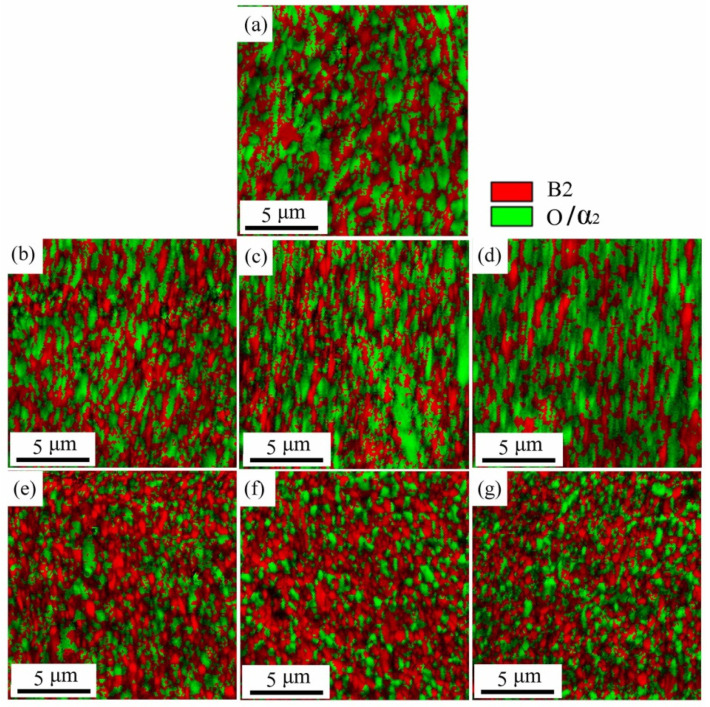
IQ + Phase figure of BM of Ti_2_AlNb alloy spun workpiece formed at different passes: (**a**) initial welded tube, (**b**) pass 1st, (**c**) pass 2nd, (**d**) pass 3rd, (**e**) pass 4th, (**f**) pass 5th, (**g**) pass 6th.

**Figure 7:**
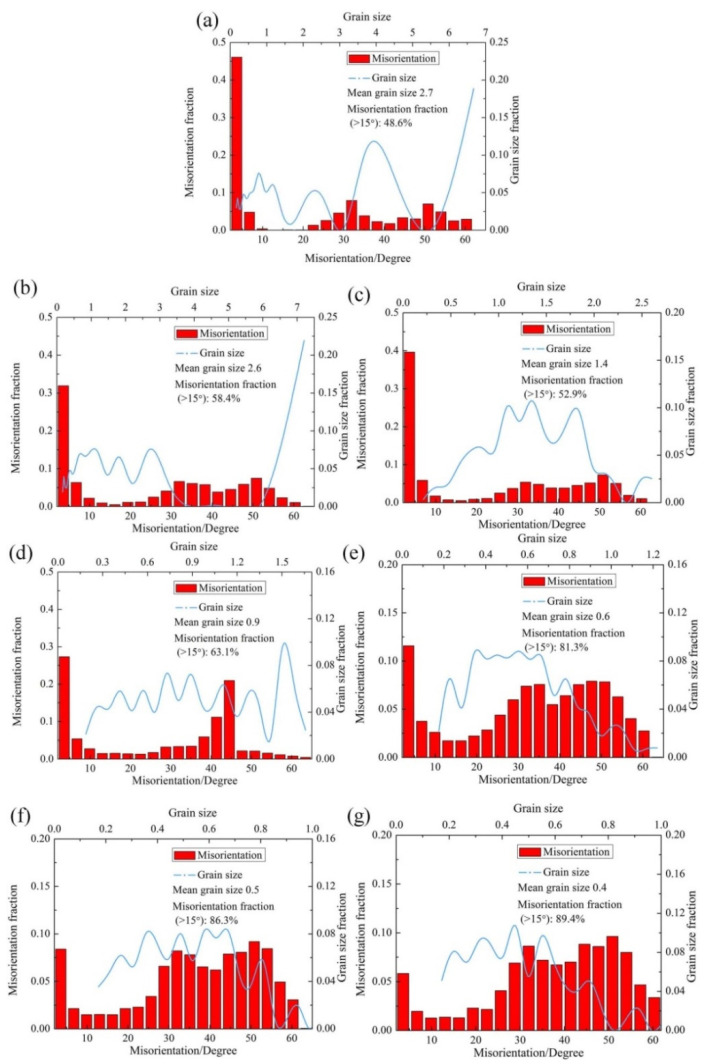
IQ + Phase figure of BM of Ti_2_AlNb alloy spun workpiece formed at different passes: (**a**) initial welded tube, (**b**) pass 1st, (**c**) pass 2nd, (**d**) pass 3rd, (**e**) pass 4th, (**f**) pass 5th, (**g**) pass 6th.

For further revealing the deformation mechanism in BM of the spun tube, the corresponding microstructures are characterized using EBSD and TEM in this study. Figure 6 presents the IQ + Phase maps (Image Quality + Phase) of the spun BM formed at different passes. Figure 7 shows the statistical data of B2 phase grain size as well as misorientation fraction. Actually, it is hard to discern α_2_ and O phase based on EBSD result because of little difference between each other in crystal structure [32]. Thus, they were integrated as O phase, and marked in green color. During 1st to 3rd pass spinning (*ψ*_t_ = 20–49%), the B2 and O phases were elongated gradually since the axial flow of metal dominated plastic deformation of tube spinning, and the high-angle boundary (HAB, misorientation > 15°) fraction of B2 phase was less than 63.1%, see Figure 6b–d. Beyond 4th pass spinning (*ψ*_t_ = 59%), amounts of spheroidized recrystallization grains were formed, and the HAB fraction was higher than 81.3%, which was consistent with the analysis result of B2 phase grain morphology, see Figure 6e–g and Figure 7e–g. With the increase in thinning ratio, the LAB (misorientation < 15°) usually evolved into HAB by absorbing dislocations. Due to severe plastic deformation and dynamic recrystallization during hot flow spinning, the B2 phase grain size dropped from 2.7 μm to 0.4 μm after six-pass spinning (*ψ*_t_ = 74%), as shown in Figure 6 and Figure 7.

**Figure 8:**
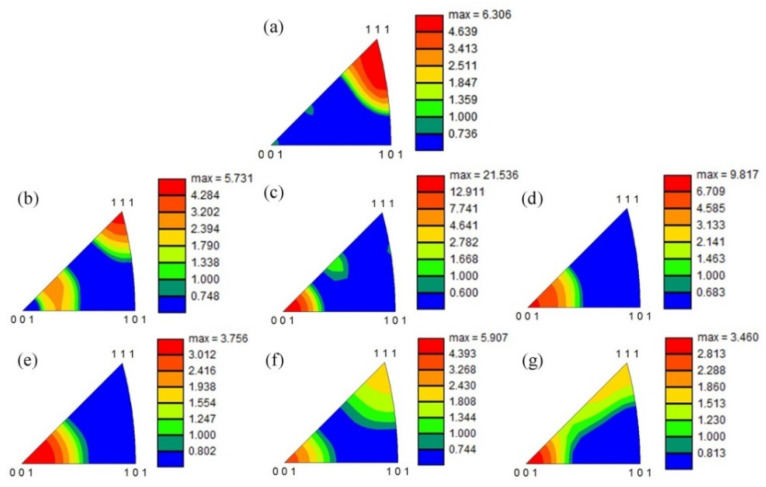
Inverse Pole Figure of B2 phase in normal direction (ND) of the spun workpiece formed at different passes: (**a**) initial welded tube, (**b**) pass 1st, (**c**) pass 2nd, (**d**) pass 3rd, (**e**) pass 4th, (**f**) pass 5th, (**g**) pass 6th.

**Figure 9:**
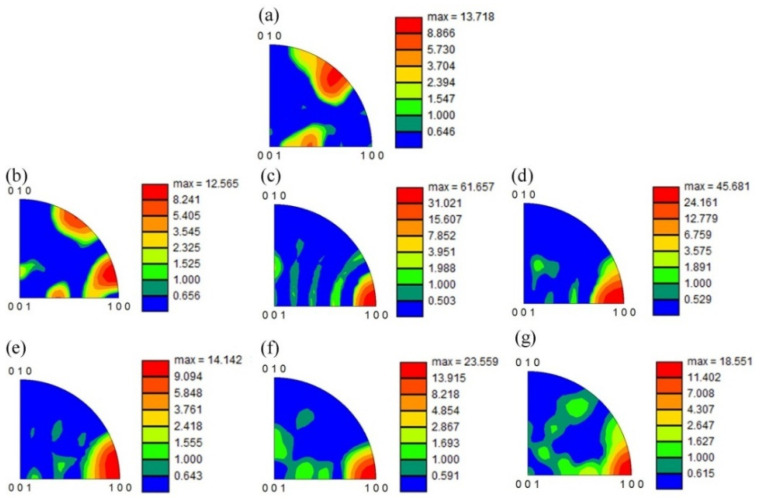
Inverse Pole Figure of O phase in normal direction (ND) of the spun workpiece formed at different passes: (**a**) initial welded tube, (**b**) pass 1st, (**c**) pass 2nd, (**d**) pass 3rd, (**e**) pass 4th, (**f**) pass 5th, (**g**) pass 6th.

For the purpose of further investigating the orientation characteristics of the grains in the Ti_2_AlNb-based spun workpieces, the corresponding inverse pole figures (IPFs) of B2 and O phase were obtained based on the EBSD data, see Figure 8 and Figure 9, respectively. The B2 phase of the as-received alloy possesses a strong <111>_B2_ texture in the normal direction (ND), i.e., <111>_B2_//ND, see Figure 8a. The slip systems {110}<111>_B2_ and {112}<111>_B2_, were activated when the spinning process operated. Meanwhile, <001>_B2_ texture formed gradually in the normal direction, i.e., <001>_B2_//ND, see Figure 8b. It can be found in Table 3, all the B2 phase Schmid factors were higher than 0.4 under 0–6-pass, which demonstrated that the two slip systems had a good slip deformation capacity, see Table 3. Meanwhile, as the <001>_B2_ texture evolved, the <100>_O_ texture emerged simultaneously, see Figure 9c. As B. Shao et al. [1] reported that if the slip plane and direction of O phase were the same as B2 phase, it is favorable for O phase slip. After 5th pass spinning, the <001>_B2_ texture weakened, and the <111>_B2_ texture emerged again since dynamic recrystallization occurred evidently during the hot spinning process, see Figure 8f,g.

**Figure 10:**
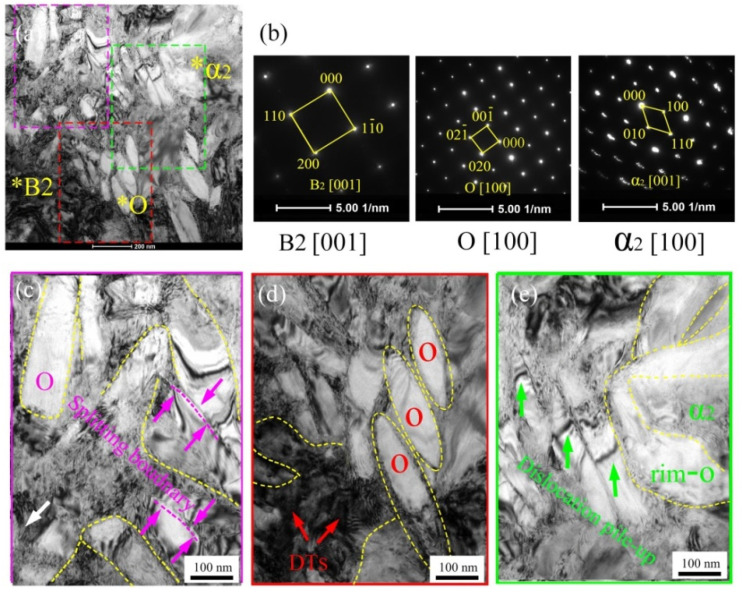
TEM micrographs of initial welded tube (*ψ*_t_ = 0%): (**a**) bright field figure, (**b**) selected area electron diffraction of (**a**), (**c**–**e**) local enlarged figure with three features of (**a**). The position marked by * corresponds to the diffraction pattern of Figure 10b.

**Figure 11:**
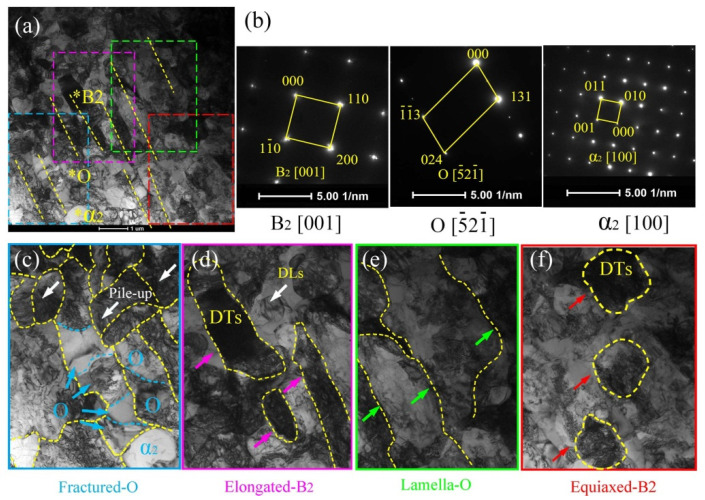
TEM result of the 3rd pass spun tube (*ψ*_t_ = 49%): (**a**) bright field figure, (**b**) selected area electron diffraction of (**a**), (**c**–**f**) local enlarged figure with three features of (**a**). The position marked by * corresponds to the diffraction pattern of Figure 11b.

**Figure 12:**
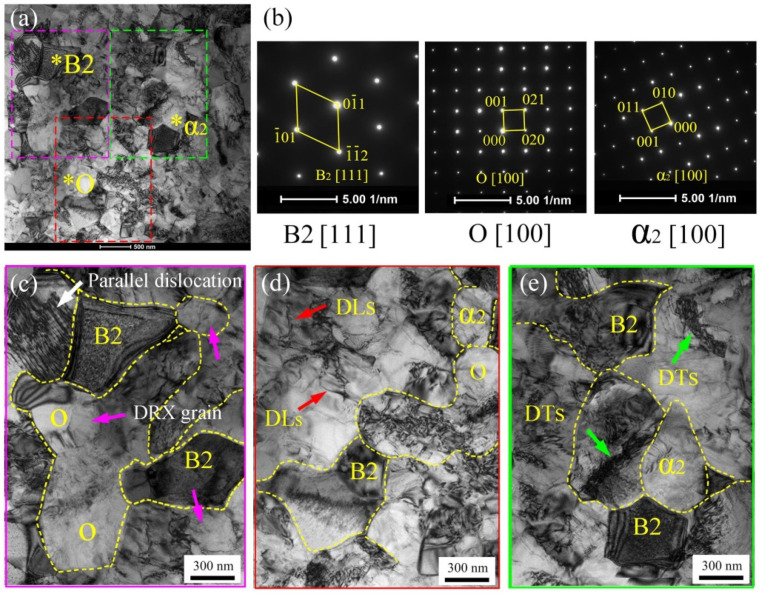
TEM result of the 6th pass spun tube (*ψ*_t_ = 74%): (**a**) bright field image, (**b**) selected area electron diffraction of (**a**), (**c**–**e**) local enlarged image with three features of (**a**). * The position marked by * corresponds to the diffraction pattern of Figure 12b.

For identifying the phase structure as well as morphology evolution of spun tube of Ti_2_AlNb alloy, TEM micrographs in BM of the initial welded tube, 3rd and 6th pass spun workpiece were shown in Figure 10, Figure 11 and Figure 12, respectively. The TEM analysis indicates that the initial microstructure of initial welded tube contained B2, O and α_2_ phase, which could be observed by the diffraction pattern of selected area, see Figure 10a,b. Three prominent features based on TEM micrographs were shown in three local enlarged images: splitting boundaries between O phase, see Figure 10c; dislocation tangles (DTs) in B2 phase, see Figure 10d; dislocation pile-up in O phase, see Figure 10e. In addition, the length and width of acicular O phase were about 200 nm and 30 nm, respectively, see Figure 10d.

Figure 11 shows the prominent deformation features (fractured O phase, Elongated B2, lamella-O, equiaxed-B2) of the 3rd pass spun tube (*ψ*_t_ =49%) based on TEM micrographs. First, the brittle O phase was fractured and crushed into granular shape, as pointed by blue arrows in Figure 11c. Second, plastic deformation mainly operated in the B2 phase matrix, and plenty of elongated B2 phases could be seen in Figure 11d, as pointed by purple arrows. Besides, a large number of dislocation lines (DLs) and dislocation tangles (DTs) could be found in B2 matrix, see Figure 11d,f. Third, lamella O phase were still could be found along the axial direction of spun tube since the moderate deformation degree could not be used to crashed O phase thoroughly, see Figure 11e. Last, plenty of equiaxed-B2 phase appeared due to partial dynamic recrystallization during hot spinning, see Figure 11f.

**Figure 13:**
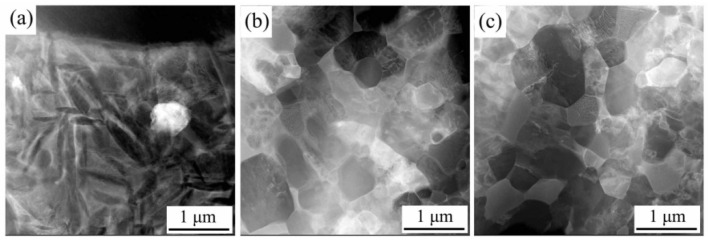
HADDF images of spun workpieces: (**a**) initial welded tube, (**b**) pass 3, (**c**) pass 6.

Figure 12 presents the TEM results of the 6th pass spun tube with the thinning ratio of 74%. The ultra-fine grains of equiaxial O, α_2_ and B2 phase can be seen in Figure 12a. The local enlarged figures of Figure 12a were shown in Figure 12c–e. The fine O phase with 500 nm average grain size distributed homogeneously, as estimated in Figure 12c. The red arrows and green arrows in Figure 12d,e illustrated the dislocation lines (DLs) and dislocation tangles (DTs), respectively. The DLs and DTs were found in equiaxial grain interior, and faded in grain boundaries, indicating that the dislocations of the spun microstructure were in an equilibrium state. Similar to other SPD processes [33,34], the grain size could be refined by dynamic recrystallization based on the high dislocation density accumulated by flow spinning [35]. In addition, see Figure 13a–c, accompanied with lenticular O phase disappeared, the B2 phase microstructure was gradually refined to 0.4 μm after six passes spinning due to the refinement mechanism dynamic recrystallization, which corresponding to the EBSD result of Section 3.2.1.

#### 3.2.2. Microstructure Evolution of FZ

**Figure 14:**
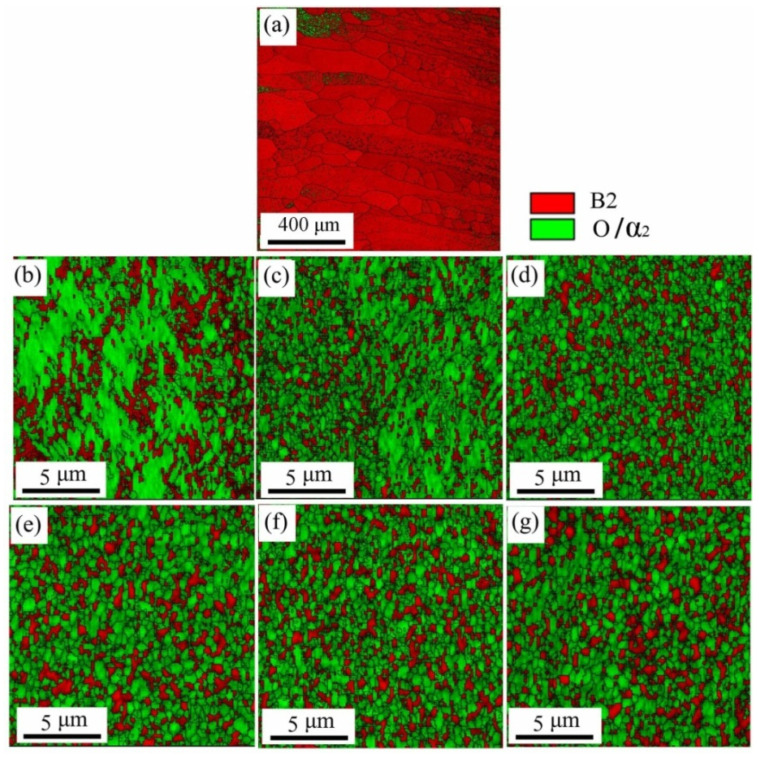
IQ + Phase figure of FZ of Ti2AlNb alloy spun workpiece formed at different passes: (**a**) initial welded tube, (**b**) pass 1st, (**c**) pass 2nd, (**d**) pass 3rd, (**e**) pass 4th, (**f**) pass 5th, (**g**) pass 6th.

Figure 14 illustrates the EBSD result of IQ + Phase maps in FZ of welded spun workpieces formed at different passes. The mean grain size of B2 phase was 10.8 μm, see Figure 14a. As the thinning ratio increased, the mean grain size decreased dramatically caused of dynamic recrystallization. Compared with BM, dynamic recrystallization took place much earlier in FZ. After 1st pass spinning, amounts of recrystallized B2 grains with diameter 2.9 μm could be found in the IQ + Phase map with the HAB fraction of 75.26 %, see Figure 14b. After 6th pass spinning, the mean grain size of B2 in FZ decreased to 0.6 μm with the HAB fraction of 83.54%, see Figure 14g.

### 3.3. Mechanical-Property Analysis during Multi-Pass Power Spinning

**Figure 15:**
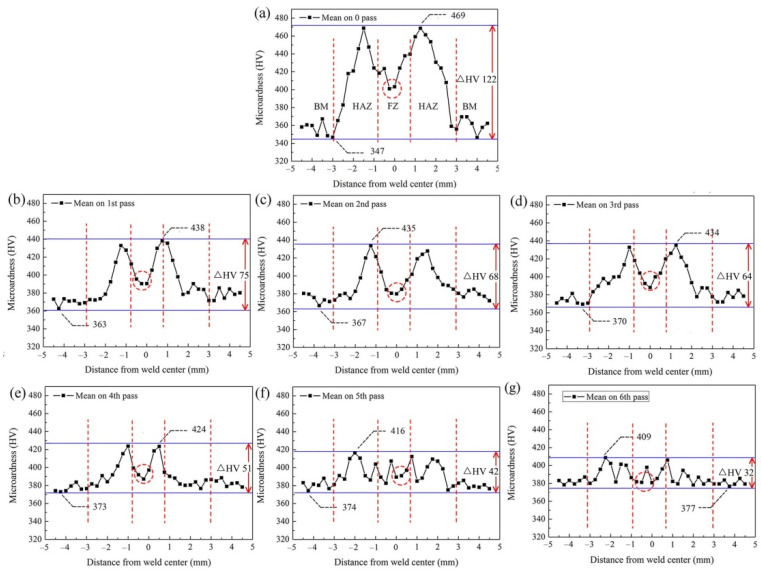
Microhardness evolution of the weld joint during 6-pass hot power spinning: (**a**) initial welded tube, (**b**) pass 1st, (**c**) pass 2nd, (**d**) pass 3rd, (**e**) pass 4th, (**f**) pass 5th, (**g**) pass 6th.

The Vickers hardness values of as-welded and post-welded spun joints were shown in Figure 15, indicating the variation from BM to FZ. Clearly, the microhardness curve of as-welded joint illustrated a bimodal feature. The microhardness profile was closely related to the phase compositions [12]. After six-pass spinning, the average microhardness in FZ dropped from HV 410 to HV 390, corresponding to the phase evolution of B2 → B2 + O + α_2_ phases. Different from FZ, a markedly decrease in microhardness was observed in HAZ, and the peak value dropped from HV 469 to HV 409. Besides, the microhardness of BM increased from HV 347 to 377, mainly due to the effect of fine grain strengthening. The difference of the microhardness of BM, HAZ and FZ tended to be minimized with the increase in thinning ratio during hot flow spinning. It can be found in Figure 14, after six-pass spinning the microhardness fluctuation ΔHV dropped to 32 from 122 of the as-welded joint, which should be ascribed to the phase composition and microstructure homogenization caused by multi-pass power spinning.

The tensile strength of the spun workpieces of base metal in axial direction and tangential direction were listed in Table 4. After six-pass spinning, the UTS (ultimate tensile strength) in axial/tangential direction was increased to 1245/1299 MPa from 1206/1010 MPa of the as-received rolled sheet, respectively, mainly due to the effect of fine grain strengthening. Besides, another factor should not be ignored, i.e., the reduction in B2 phase content from 55.4% of as-received sheet to 44.1% of spun tube in final pass, as well as the increase in O phase content from 31.4% to 43.8% correspondingly. Shao et al. reported that the B2 phase deformation ability was much better than that of O and α_2_ phases since the BCC microstructure had more slip systems [1]. On the other hand, after six-pass spinning, the anisotropy of mechanical properties was reversed, the highest UTS changed from the axial direction to the tangential direction, which should be attributed to the co-effect of texture transformation and O phase morphology evolution caused by multiple-pass spinning.

Furthermore, due to the co-effect of high spinning temperature, heat affect and complicated stress condition, the aspect ratio of the O phase platelets (AROP) reduced from 6.8 to 1.4 when the six spinning passes were finished. Jiao et al. reported that the high AROP was beneficial for improving ductility [36]. Therefore, after six-pass spinning, the total elongation in axial/tangential direction was decreased to 8.1%/7.3% from 14.3%/14.6% of the as-received alloy, respectively, mainly due to the reduction in AROP and B2 content as well as work hardening. However, the multi-pass power spinning process could homogenize the microstructure and improve the mechanical property of welded tube, indicating the feasibility for manufacturing high-performance thinned walled tubular workpieces of Ti_2_AlNb-based alloy in lower cost and higher efficiency.

## 4. Conclusions

The multi-pass power spinning experiment was conducted successfully using welded tube blank of Ti-22Al-24Nb-0.5Mo alloy with total thinning reduction 74% under the temperature 950±30 °C controlled by electro-magnetic induction equipment. The microstructure evolution and mechanical property of six pass hot spun tubes were systematically investigated, and the main conclusions were summarized as follows:

(1) During 1st–3rd pass spinning, i.e., the thinning ratio of 20–49%, the B2 phase in BM were stretched along with axial direction gradually, when <111>_B2_//ND texture weakened and <001>_B2_//ND texture occurred gradually. After 6th pass spinning, both <001>_B2_//ND texture and <111>_B2_//ND texture co-existed in BM, due to the texture weakness caused by B2 phase recrystallization during hot power spinning.

(2) The small equiaxed B2 grains were generated at the positions of severe lattice distortion, induced by dynamic recrystallization. In the final pass, the thinning ratio reached 74%, and the B2 phase grain size was refined to 0.4/0.6 μm from 2.7/10.8 μm of the as-received BM/FZ, respectively.

(3) The values of microhardness of BM, HAZ and FZ tended to be similar with the increase in thinning ratio during hot flow spinning, and the microhardness fluctuation ΔHV dropped to 32 from 122 of the as-welded joint, which should be attributed to the homogenization of phase composition and microstructure caused by multi-pass power spinning. Besides, the UTS (ultimate tensile strength) in axial/tangential direction was increased to 1245/1299 MPa from 1206/1010 MPa of the as-received rolled sheet, respectively, mainly due to the effect of fine grain strengthening. Meanwhile, the total elongation in axial/tangential direction was decreased to 8.1%/7.3% from 14.3%/14.6% of the as-received alloy, respectively, mainly due to the decline of AROP (aspect ratio of the O phase platelets) and B2 content as well as work hardening.

## Figures and Tables

**Table 1:** Main process parameters of hot power spinning.

Forming Parameters	Values
Initial tube blank length, *L* (mm)	80
Initial tube blank thickness, *t*_0_ (mm)	6
Mandrel diameter *D*_m_ (mm)	100
Roller diameter, *D*_r_ (mm)	200
Roller feed rate, *f* (mm/r)	1
Front angle of roller, *α*_ρ_ (deg)	20
Fillet radius of roller, *r* (mm)	5
Rotation speed of mandrel, *n* (rpm)	200
Reduction, *ψ*_t_ (%)	20, 36, 49, 59, 67, 74
Thickness of spun workpiece, mm	6.0, 4.8, 3.84, 3.07, 2.46, 1.97, 1.58
Roller number	2
Temperature (°C)	950 ± 30

**Table 2:** Area fraction of α_2_, O and B2 phase in the welded joints (%). (Measured by the Image-Pro Plus software).

	FZ			HAZ Close to BM			BM		
	B2	α_2_	O	B2	α_2_	O	B2	α_2_	O
As-welded	95.5 ± 4.5	4.5 ± 0.2	/	81.5 ± 4.1	12.8 ± 0.7	5.7 ± 0.3	55.4 ± 2.8	13.2 ± 0.7	31.4 ± 1.6
Pass 1	42.6 ± 2.8	10.3 ± 0.2	47.1 ± 0.9	48.7 ± 2.4	12.5 ± 0.7	38.8 ± 1.9	52.3 ± 2.8	13.3 ± 0.7	34.4 ± 1.7
Pass 2	41.7 ± 2.8	10.7 ± 0.4	47.6 ± 1.3	46.6 ± 2.3	12.5 ± 0.7	40.9 ± 2.1	49.1 ± 2.5	12.8 ± 0.7	38.1 ± 1.9
Pass 3	40.4 ± 2.1	11.5 ± 0.4	48.1 ± 1.3	45.2 ± 2.3	12.2 ± 0.7	42.6 ± 2.1	48.1 ± 2.5	12.8 ± 0.7	39.1 ± 2.1
Pass 4	39.1 ± 2.1	11.7 ± 0.4	49.2 ± 1.4	42.6 ± 2.1	12.2 ± 0.7	45.2 ± 2.3	44.3 ± 2.2	12.5 ± 0.7	43.2 ± 2.1
Pass 5	38.5 ± 1.9	11.9 ± 0.5	49.6 ± 1.4	42.3 ± 2.1	12.1 ± 0.6	45.6 ± 2.3	44.2 ± 2.2	12.3 ± 0.7	43.5 ± 2.2
Pass 6	38.4 ± 1.8	12.1 ± 0.5	49.5 ± 1.5	42.2 ± 2.1	12.1 ± 0.6	45.7 ± 2.3	44.1 ± 2.2	12.1 ± 0.6	43.8 ± 2.2

**Table 3:** Schmid factors of the two main slip systems of B2 phase in axial and tangential direction.

Slip System	Pass						
	0	1	2	3	4	5	6
{110}<111>_B2-axial direction_	0.43	0.44	0.46	0.43	0.47	0.42	0.46
{110}<111>_B2-tangential direction_	0.47	0.45	0.47	0.45	0.47	0.45	0.47
{112}<111> _B2-axial direction_	0.47	0.46	0.47	0.46	0.46	0.44	0.46
{112}<111> _B2-tangential direction_	0.47	0.49	0.47	0.48	0.47	0.48	0.46

**Table 4:** Comparison of mechanical properties between the 6-pass spun tube in axial and tangential direction (Base metal).

Pass	BM-Axial		BM-Tangential	
	UTS/MPa	Total Elongation %	UTS/MPa	Total Elongation %
0	1206 ± 16	14.3 ± 0.7	1010 ± 46	14.6 ± 0.7
1	1212 ± 26	13.1 ± 0.7	1043 ± 27	11.9 ± 0.6
2	1224 ± 16	9.4 ± 0.5	1109 ± 30	9.0 ± 0.5
3	1237 ± 20	9.4 ± 0.5	1152 ± 38	9.0 ± 0.5
4	1242 ± 15	9.4 ± 0.5	1196 ± 28	7.5 ± 0.4
5	1252 ± 8	9.5 ± 0.5	1247 ± 70	7.9 ± 0.4
6	1245 ± 26	8.1 ± 0.4	1299 ± 40	7.3 ± 0.4
